# Right subclavian artery injury during catheter insertion into the right internal jugular vein treated with endovascular stent graft placement after balloon occlusion test: A case report

**DOI:** 10.1016/j.radcr.2024.03.025

**Published:** 2024-04-13

**Authors:** Yuto Tamaoki, Ryo Kamidani, Hideshi Okada, Takahito Miyake, Kodai Suzuki, Takahiro Yoshida, Keisuke Kumada, Shozo Yoshida, Shinji Ogura

**Affiliations:** a Advanced Critical Care Center, Gifu University Hospital, 1-1, Yanagido, Gifu city, Gifu 501-1194, Japan; b Abuse Prevention Center, Gifu University Graduate School of Medicine, 1-1, Yanagido, Gifu city, Gifu 501-1194, Japan; c Patient Safety Division, Gifu University Hospital, 1-1, Yanagido, Gifu city, Gifu 501-1194, Japan

**Keywords:** Balloon occlusion test, Central venous catheter, Cerebral infarction, Complication, Subclavian artery injury, VIABAHN stent-graft

## Abstract

Subclavian artery injuries during internal jugular vein puncture when attempting central venous catheter insertion are rare. A 60-year-old man undergoing treatment for neuromyelitis optica with paralysis and sensory loss developed a complication during catheter placement into his right internal jugular vein for plasmapheresis. His previous physician felt resistance and discontinued the procedure. The patient later developed mild dyspnea and dysphagia. Computed tomography scans indicated thrombus formation and tracheal deviation. Contrast-enhanced computed tomography scans showed right subclavian artery injury with extravasation and a large pseudoaneurysm.

Following transferal to our hospital, he was stable and asymptomatic; however, contrast-enhanced computed tomography scans showed a pseudoaneurysm located proximal to the right subclavian artery. Considering challenges with compression hemostasis and the invasiveness of open surgery, endovascular treatment was selected using a VIABAHN stent graft. A balloon occlusion test of the right vertebral artery was performed to assess stroke risk. Prophylactic embolization of the right vertebral artery, internal thoracic artery, and thyrocervical trunk were performed to prevent a type 2 endoleak. On hospital day 5, our patient showed no postoperative complications and was transferred to the referring hospital. Follow-up imaging showed the graft was intact with no pseudoaneurysm, confirming successful treatment. Endovascular treatment with a stent graft is highly effective for peripheral artery injuries. Using a balloon occlusion test to assess collateral blood flow and stroke risk is essential pretreatment, especially when a graft might occlude the vertebral artery. Balloon occlusion tests are recommended when planning treatment for iatrogenic and other types of subclavian artery injuries.

## Introduction

Arterial puncture has been reported to occur in 6.3%-9.4% of internal jugular vein catheterizations and in 3.7% of subclavian vein catheterizations; however, subclavian artery puncture during central venous catheter (CVC) insertion into the internal jugular vein is an extremely rare complication [1,2]. This serious complication is challenging to detect as it is deep in relation to the puncture point; thus, application of direct pressure and obtaining hemostasis are challenging. We encountered a case of iatrogenic right subclavian artery injury owing to a puncture of the right internal jugular vein (RIJV) during CVC insertion. The injury was successfully repaired by physicians from different disciplines.

### Case summary

A 60-year-old man with no history of arteriosclerosis or other medical history, medications, or allergies was undergoing treatment at another hospital for neuromyelitis optica with bilateral lower extremity paralysis and sensory deficits below the papillary level. Blood test results taken during hospitalization showed no abnormalities in hemostasis or coagulation ability. On day 10 postadmission, an attempt was made to insert a CVC into the RIJV to perform plasmapheresis. The guidewire was inserted, but the physician felt resistance while inserting the dilator. The patient experienced pain in the right arm and developed a right-sided neck swelling, causing the physician to abandon the insertion of the CVC and attempt compression hemostasis. The CVC was inserted into the right femoral vein and plasmapheresis was performed.

**Fig. 1:**
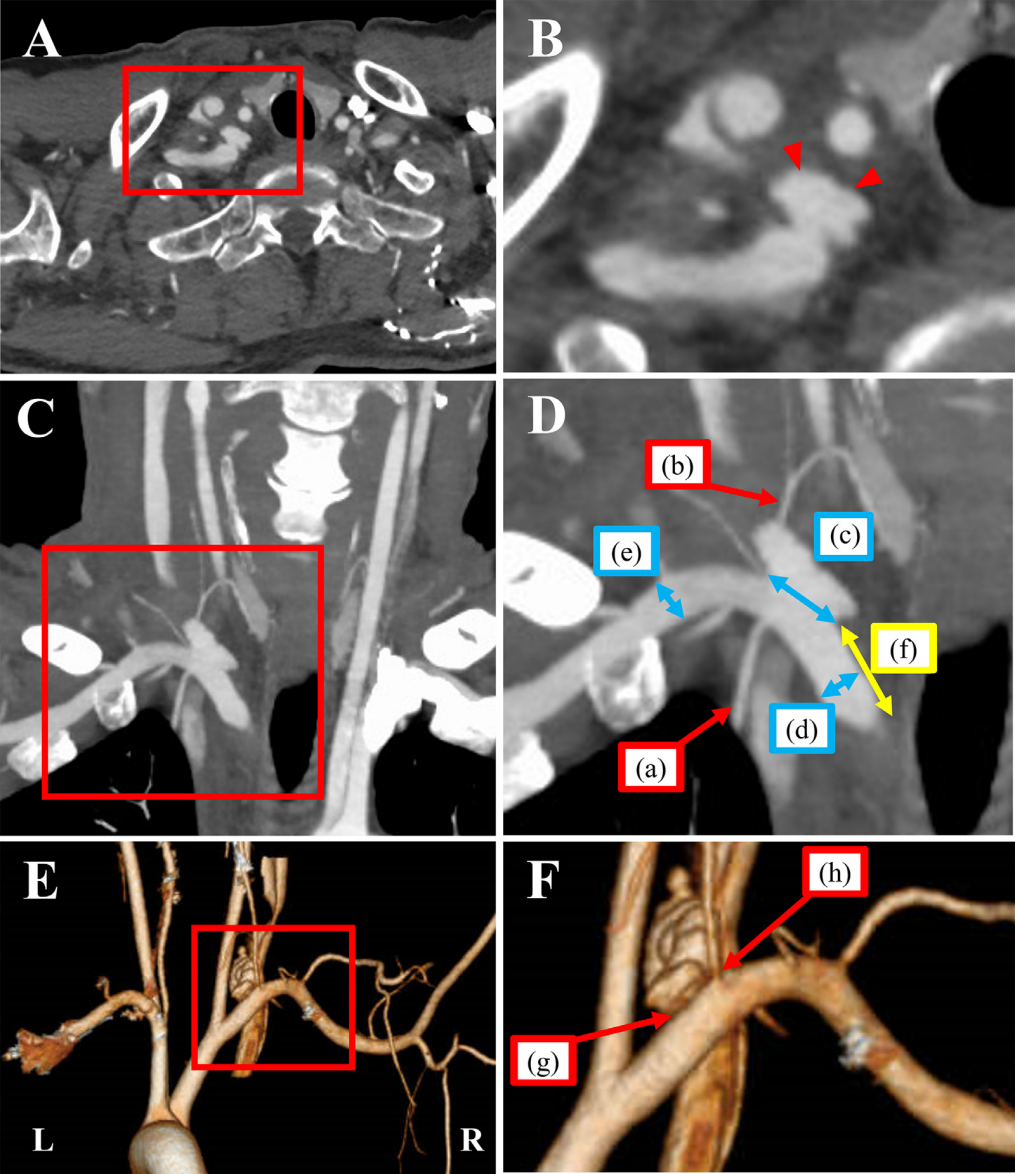
Contrast-enhanced computed tomography (CT) scan taken by the previous physician. Contrast-enhanced CT findings, revealing (A, B) a large saccular pseudoaneurysm (red arrow). (C, D) The pseudoaneurysm, located 13.0 mm from the right subclavian artery bifurcation, had an inlet diameter of 3.0 mm. The proximal and distal diameters of the pseudoaneurysm were 8.0 mm and 6.0 mm, respectively (a: right internal thoracic artery, b: right thyrocervical artery, c: 3.0 mm, d: 8.0 mm, e: 6.0 mm, f: 13.0 mm). (E, F) A dorsal view of the 3D reconstruction of the contrast-enhanced CT image showed the proximity of the pseudoaneurysm and the right vertebral artery (G: pseudoaneurysm, H: right vertebral artery).

Subsequently, the patient complained of mild dyspnea, dysphagia, and decreased oxygenation. Plain computed tomography (CT) was performed, revealing thrombus formation in the RIJV and tracheal deviation. While the patient's general condition appeared to be good, he remained hospitalized for observation. The following day, a contrast-enhanced CT of the neck revealed an injury to the right subclavian artery, with extravasation and a large pseudoaneurysm (Fig. 1A and B). The patient was then transferred to our hospital.

Following admission to our institution, his physical examination findings were as follows: respiratory rate, 15 breaths per minute; saturation of percutaneous oxygen, 100% under nasal cannula (2 L/min); heart rate, 78 beats per minute; normal hemodynamic parameters (blood pressure, 100/66 mmHg without the use of catecholamine agonists), and body temperature, 36.4 °C. The right side of his neck was slightly swollen. Laboratory blood test results on admission showed no significant abnormalities. Contrast-enhanced CT scans showed a pseudoaneurysm located proximal to the right subclavian artery with an entry diameter of 3.0 mm (Fig. 1C). The right vertebral artery bifurcated from the dorsal inlet, and the right internal thoracic and right thyroid carotid arteries bifurcated from the peripheral entry. The length of the right subclavian artery, from its origin to the entry of the pseudoaneurysm, measured 13.0 mm, and its proximal and distal vessel diameters measured 8.0 mm and 6.0 mm, respectively (Fig. 1D). Additionally, the vertebral arteries were co-dominant with a vascular diameter of 5.6 mm bilaterally.

**Fig. 2:**
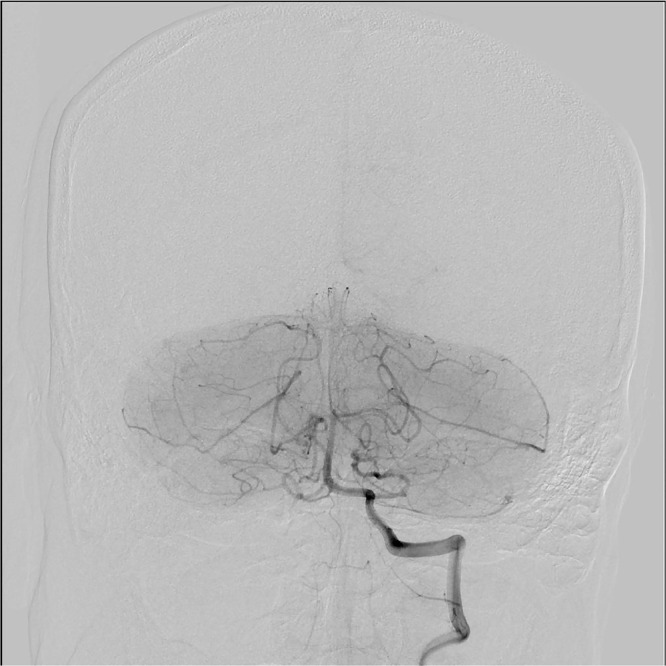
Angiography findings. The left vertebral angiography confirmed blood flow to the right posterior circulation and validated the arterial circle of Willis.

**Fig. 3:**
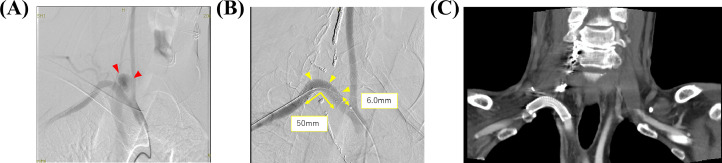
Angiography findings. (A) the cystic contrast area just after the right subclavian artery bifurcation and (B, C) the 6.0 mm distance from the bifurcation of the right subclavian artery and the right common carotid artery to the proximal endovascular stent. The total length was 50 mm and no endoleak or positional deviation was observed after implanting the stent graft.

On day 3, a vascular surgeon surgically exposed the right brachial artery and placed a 6 Fr sheath. A neurosurgeon placed a 4 Fr sheath into the right femoral artery and performed a balloon occlusion test (BOT) of the right vertebral artery (Fig. 2). Consequently, the collateral vessels were shown to be adequate, and the neurosurgeon performed coil embolization on the right vertebral artery. A radiologist embolized the right internal thoracic artery and thyrocervical trunk. The 6 Fr sheath implanted in the right brachial artery was replaced with an 8 Fr sheath and a VIABAHN (Gore, Flagstaff, AZ) stent graft (SG) (9 mm × 5 cm) was implanted. The pseudoaneurysm was not observed on the final angiogram, and the vascular surgeon closed the exposed right brachial artery and completed the surgery (Fig. 3A-C).

The patient had an uneventful clinical course postoperatively and, on hospital day 5, he was transferred back to the referring hospital. CT angiography (CTA) was performed on day 10 at the referring hospital, showing adequate contrast flow through the SG with normal filling of the distal vessels, and no findings of pseudoaneurysm, rebleeding, or endoleak. As this was a case of hemorrhage, the patient did not require antithrombotic drugs postoperatively. In terms of a future management plan, the patient will continue to be monitored clinically and with CTA every 6 months for 12 months, then followed by Doppler ultrasonography using the Duplex method annually, all of which will be undertaken at the referring hospital.

## Discussion

Here, we report a case of iatrogenic injury to the right subclavian artery, which occurred while attempting to insert a CVC into the RIJV. Our patient was treated with a VIABAHN SG placed after having performed a BOT to determine the effect of the right vertebral artery covering. Endovascular treatment is effective in peripheral vascular injuries due to CVC insertion in areas where compression hemostasis is challenging, provided that the efficacy of the collateral blood vessels is evaluated beforehand.

Complications occur in >15% of central venous catheterizations, and between 5% and 19% of these complications are known to be mechanical [1]. Among these complications, arterial puncture rates are low (approximately ranging from 4% to 9%), and arterial puncture at different target points from the puncture point is even less frequent, with no specific figures reported. Generally, subclavian and axillary artery injury rates have been reported to range from 5% to 10% in terms of vascular injuries in civilian trauma centers, mostly from penetrating trauma [3]. Yoshida et al. reported a case of CVC misplacement into the right subclavian artery during general anesthesia for emergency thoracic endovascular aortic repair of a traumatic descending dissection when insertion was attempted from the RIJV [4]. An intravascular stent was inserted and then removed because a catheter >7 Fr in size was mistakenly inserted at a site where compression hemostasis was difficult. In similar cases, CVCs incorrectly inserted in subclavian arteries have been safely removed using an angio-seal or intravascular stent [5,6]. A case of open subclavian artery repair and RIJV ligation in addition to sternotomy catheter removal has also been reported [7].

Generally, when a CVC is misplaced, especially when a hematoma has formed, removal is performed with compression hemostasis, endovascular treatment, or surgery [8]. In this case, the access site was the right subclavian artery; therefore, compression hemostasis was expected to be difficult, and endovascular treatment was selected based on the degree of invasiveness. Prior to the widespread use of endovascular treatment, the standard approach to subclavian artery injury was open thoracotomy. Anatomically, the subclavian region is difficult to approach, and in some cases, a median sternotomy alone may not be sufficient, requiring a posterolateral or supraclavicular incision [3]. In an earlier but large review of subclavian artery and vein injuries, 11.8% of patients were reported to have died following open repair or ligation, and 4.8% died after excluding emergencies [9]. Despite the highly invasive nature of this procedure, vascular surgeons were on standby if the treatment was unsuccessful.

A surgeon's experience plays a major role in the occurrence of complications during CVC placement; if the physician has previously placed ≥50 CVCs, the rate of complications during insertion is approximately halved [10]. In this case, while the experience level of the physician who first attempted to insert the CVC is unknown, that physician was a resident doctor with <1 year of experience, and the teaching assistant involved was an attending physician with >15 years of experience. To avoid mechanical complications that may arise during insertion of a CVC into the internal jugular or subclavian veins, it is recommended to: (i) keep the patient's head low (in the so-called Trendelenburg position) with their shoulders positioned posteriorly, (ii) confirm anatomic relationships and vessel patency at the time of preparation, (iii) plan the appropriate anatomic level at which the venipuncture is to be attempted, (iv) perform real-time observation with ultrasound, and (v) avoid multiple attempts at the same site [11,12]. When access is attempted through the subclavian artery, a 6-fold (4%-24%) increase in the complication rate has been reported when the needle is threaded more than once [10]. Hematoma formation and arterial injury can occur during puncture, dilation, or catheter insertion. If mechanical complications occur, the incorrectly positioned catheter should not be removed. Additionally, physicians from multiple departments should be involved in planning treatment strategies. Where possible, compression hemostasis should be attempted first.

We report a case of endovascular treatment following BOT results, conducted to repair an accidental puncture of the right subclavian artery during the insertion of a CVC into the RIJV. The manufacturer's instructions state that, when using the VIABAHN SG, maintaining a neck length of at least 20 mm is important to avoid rebleeding and type 1 endoleaks. In some cases, securing neck length in the visceral arteries, and even in the subclavian artery, may be challenging as the presence of major branches may make it impossible to achieve the appropriate distance or the branch arteries may also need to be covered. Unlike aortic SGs, type 2 endoleaks of peripheral arterial SGs are challenging to treat. Therefore, if such leaks occur or are anticipated, prophylactic embolization of the branch should be considered prior to implanting the SG [13]. In this case, prophylactic embolization was performed on the right internal thoracic and right thyrocervical arteries, which are branches of the right subclavian artery.

Previous reports of emergency endovascular treatment of upper and lower arterial bleeding with VIABAHN SG implantation have shown high technical and clinical success rates (97%-100%), with somewhat variable SG patency rates of 68–100% at different follow-up periods [14], [15], [16], [17], [18], [19]. In a study of endovascular treatment of popliteal artery aneurysms using HEMOBAHN/VIABAHN SG implantation, perioperative complications occurred in 1.6% of cases and consisted of access site hematoma or acute luminal thrombosis [19]. When SGs are used in patients with fungal pseudoaneurysms or sepsis, the risk of rebleeding, endoleak, or SG occlusion may be higher due to the spread of inflammation in the vessel wall. In such cases, it is preferable to use antithrombotic and antibiotic therapy, and avoid using an undersized SG [18]. However, hemostasis is more important in bleeding cases, and antithrombotic therapy is not always essential. In this case, antithrombotic therapy was not used as the bleeding was symptomatic. We consider that our patient was not at high risk of thrombus occlusion as he had no concurrent infections and no predisposition to thrombus formation; however, we were unable to evaluate the long-term outcome as we had requested that follow-up be undertaken at the referring hospital.

Furthermore, the VIABAHN stent graft itself may have covered the right vertebral artery and caused cerebellar infarction; therefore, we performed a BOT on the right vertebral artery prior to endovascular treatment. The BOT is a method used to evaluate the effectiveness of cranial medial collateral blood circulation in maintaining perfusion in the affected vascular territory during parent vessel occlusion (PVO). PVO is often required for wide-neck giant aneurysms, pseudoaneurysms, traumatic vascular injury, carotid artery effusion, and carotid artery fistula. A previous study reported that if PVO is performed without a prior BOT, there is a 26% risk of patients having a cerebral infarction, with a higher risk of ischemia compared with 13% who undergo a BOT [20]. In actual BOTs, a 6 Fr sheath is inserted into the femoral artery, preferably on the healthy side, and 70-100 U/kg of unfractionated heparin is administered intravenously to prolong the activated clotting time to approximately 2.5 times that at baseline. A 4 Fr diagnostic catheter is used for anteroposterior and lateral projection of the 4 cervical and cerebral angiographies. The balloon is placed at or near to the predicted occlusion site and inflated for a total of 30 minutes, during which time the operator performs a neurological examination every 5 minutes [21]. Neurologic evaluation methods include clinical examination, angiographic assessments, cut indentations, induced hypotension, perfusion scans, transcranial Doppler ultrasonography, and neurophysiological monitoring, but the main parameters are clinical examination and angiographic assessments [21]. If a patient is under local anesthesia, the patient should be asked questions throughout the procedure to assess memory, speech, movement, sensation, and calculation skills. Some physicians, especially in the case of general anesthesia, will puncture the contralateral femoral artery and contrast the vertebral artery or contralateral internal carotid artery (ICA) if the ICA is the target [21]. In such cases, the venous phase assessment is useful as a BOT, based on the assumption that if the contrast effect in the venous phase is symmetrical under balloon occlusion of the parent vessel, there will be sufficient collateral blood vessels to tolerate PVO. In this case, we confirmed that the collateral blood flow was adequate prior to placing the SG immediately after the right subclavian artery bifurcation. Treatment was completed without complications arising from insufficient cerebral blood flow.

## Conclusion

Endovascular treatment using an artificial blood vessel is an effective option in cases of injury to the right subclavian artery after an attempt to insert a CVC. However, evaluating the risk of cerebral infarction beforehand is important if the vertebral artery is covered. In cases where the proximal subclavian artery is involved, physicians need to consider evaluating the efficacy of collateral circulation using a BOT.

## Declarations

**Ethics approval and consent to participate:** In Japan, approval from an ethics committee is not required to report cases. This case was reported in accordance with the ethical guidelines for medical and health research involving human subjects established by the Japanese government.

## Availability of data and materials

The datasets used and/or analyzed in the current study are available from the corresponding author upon reasonable request.

## Declaration of generative AI and AI-assisted technologies in the writing process

None.

## Authors’ contributions

Patient treatment: YT, RK, HO, TM, KS, TY, KK, SY, and SO. Study concept and design: YT and RK. Drafting and critical revision of the manuscript: YT, RK, and HO. Approval of the final manuscript: YT, RK, HO, TM, KS, TY, KK, SY, and SO.

## Patient consent

Written informed consent was obtained from the patient for publication of this case report and accompanying images.
